# Computation of the p*K*_a_ Values of Gallic Acid and Its Anionic Forms in Aqueous Solution: A Self-Similar Transformation Approach for Accurate Proton Hydration Free Energy Estimation

**DOI:** 10.3390/molecules30030742

**Published:** 2025-02-06

**Authors:** Marcin Molski

**Affiliations:** Department of Quantum Chemistry, Faculty of Chemistry, Adam Mickiewicz University of Poznań, ul. Uniwersytetu Poznańskiego 8, 61-614 Poznań, Poland; mamolski@amu.edu.pl

**Keywords:** DFT method, gallic acid, multi-step deprotonation, proton hydration, Gibbs energy, p*K*_a_ descriptor, selectivity, reactivity

## Abstract

The Gibbs free energies of gallic acid (GA) and its anionic forms in aqueous solution were computed utilizing density functional theory (DFT) at the LSDA, M062X, B3LYP/QZVP levels, in conjunction with the SMD solvation model. The p*K*_a_ values corresponding to the four-step deprotonation of GA were determined through a non-linear self-similar transformation expressed as, p*K*_a_ = a⋅p*K*_a_(the)^c^ which establishes a link between theoretical and experimental p*K*_a_ values. This approach replaces the previously employed linear relationship, p*K*_a_ = a⋅p*K*_a_(the) + b. The proposed model demonstrates high accuracy in reproducing the experimental p*K*_a_1 = 4.16 ± 0.02, p*K*_a_2 = 8.55 ± 0.01, p*K*_a_3 =11.40 ± 0.10, p*K*_a_4 =12.8 ± 0.40 values of GA, with a standard error (SE) of 0.045 and a mean absolute error (MAE) of 0.019 in p*K*_a_ unit. Furthermore, it facilitates the precise determination of the Gibbs free energy of the proton hydration, yielding ∆G(H^+^)_aq_ = 259.4272(75) [kcal mol^−1^]. This result conforms acceptably with the experimental value of ∆G(H^+^)_aq_ = −259.5 [kcal mol^−1^].

## 1. Introduction

**Figure 1:**
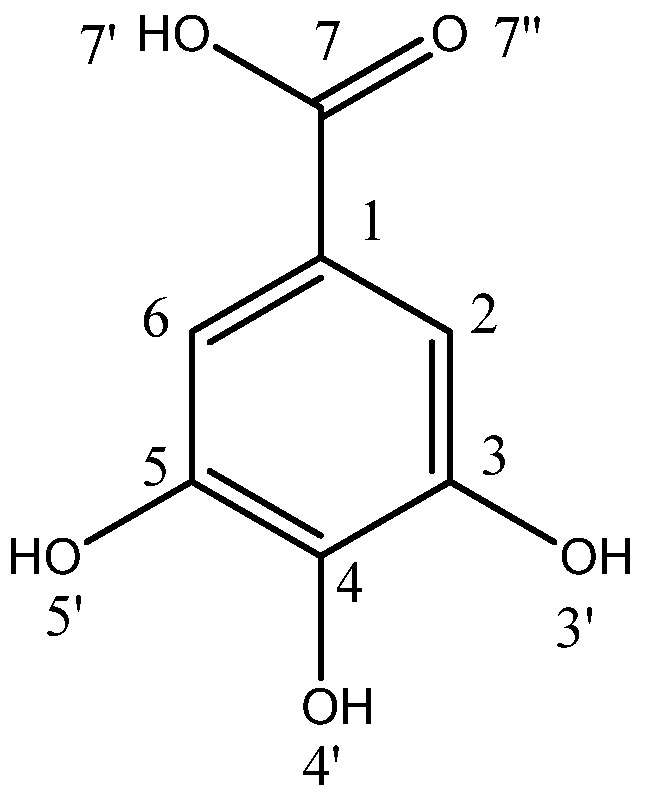
Gallic acid, 3,4,5-trihydroxybenzoic acid.

Deprotonation represents a fundamental chemical reaction that occurs across both biological [1,2] and abiotic [2,3,4] systems, thereby representing a significant research area focused on molecular reactivity and selectivity [3,4]. Monocarboxylic acids [5,6], along with their combinations with phenols and polyphenols [7,8], are particularly compelling subjects of study due to the presence of multiple reactive carboxyl and hydroxyl groups that engage actively in the deprotonation process. Experimental and theoretical analyses of protonation strive to assess the reactivity of each active group, identifying the sequential order of proton removal from the base molecule and evaluating the relative susceptibility of each group to deprotonation [3,4,9]. The chemical reactivity of these compounds is largely influenced by the electrophilic nature of the carboxylic moiety, as well as the resonance stabilization that occurs upon proton dissociation. These factors jointly contribute to both the acidity and predominant chemical reactivity of the carboxylic and phenolic groups. They can be numerically (quantitatively) characterized by the parameter p*K*_a_, which stands for the negative base-10 logarithm of an acid dissociation constant in a specific solvent, usually of a hydrophilic nature facilitating the proton detachment. Experimentally and theoretically determining the p*K*_a_ values of the compounds, especially in aqueous solution, is essential for a comprehensive understanding of numerous chemical processes and provides insight into the deprotonation state of a molecule within a specific solvent environment. Theoretical approaches to calculating p*K*_a_ parameters have garnered significant interest, particularly for applications involving molecules that have not yet been synthesized, those for which experimental determination of p*K*_a_ is challenging, and larger molecules where local environmental factors alter the intrinsic p*K*_a_ values. An example of the latter includes certain amino acids incorporated within polypeptide chains [2]. Achieving chemical accuracy in p*K*_a_ calculations is inherently challenging, as an error of 1.36 [kcal mol^−1^] in the change in free energy associated with deprotonation in a solvent corresponds to a deviation of 1 p*K*_a_ unit [2]. In this regard, gallic acid (GA, Figure 1) is among the most extensively studied compounds [3,4,9] with respect to selectivity and functional group activity, owing to its four active hydrogens in carboxyl moiety and hydroxyl groups attached to the benzene ring.

**Figure 2:**
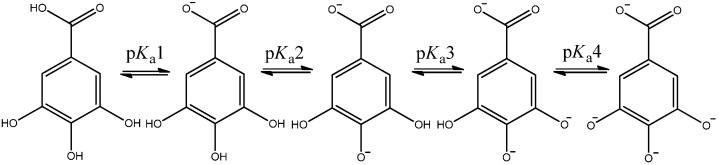
Four-step deprotonation of gallic acid and related p*K*_a_ parameters.

Research has demonstrated [3,4] that the deprotonation sequence in GA in water solution follows the order O_7′_-H, O_4′_-H, O_3′_-H, O_5′_-H, necessitating the use of distinct deprotonation descriptors: p*K*_a_1(O_7′_-H), p*K*_a_2(O_4′_-H), p*K*_a_3(O_3′_-H), p*K*_a_4(O_5′_-H) associated with the four-step deprotonation of GA presented in Figure 2.

Despite the broad utility of the p*K*_a_ descriptor, the acquisition of accurate and unique experimental data for GA remains challenging. They are commonly determined through ultraviolet–visible (UV-Vis) and Raman spectroscopy [10,11,12,13], or various analytical techniques [14]. For GA, the following experimental p*K*_a_1,2,3,4 values were reported with the standard errors if available: Set I (4.50, 7.05, 8.75, 10.25) [11], Set II (4.4 ± 0.1, 8.8 ± 0.1, 10.0 ± 0.1, 11.4 ± 0.1) [12], Set III (4.44, 8.54, 10.05, 11.30) [14], Set IV (4.16 ± 0.02, 8.55 ± 0.01, 11.40 ± 0.10, 12.8 ± 0.40) [13]. The availability of experimental p*K*_a_ values, measured with varying degrees of precision, facilitates a comparison with the theoretical results generated by different models and methods employed in the calculations. A straightforward approach to the theoretical simulation of solvent effects involves the application of various solvation models, with particular emphasis on the Conductor-like Polarizable Continuum Model (C-PCM) [15], the Integral Equation Formalism Polarizable Continuum Model (IEF-PCM) [16], and the universal Solvation Model Density (SMD) based on solute electron density [17]. They are widely employed to calculate the theoretical p*K*_a_ values of chemical compounds in solution through the relationship show below [18,19,20,21]:

(1) $$pK_{a}=\frac{{G(R}^{-}{)}_{\mathrm{sol}}-{\;G(\mathrm{RH})}_{\mathrm{sol}}\;{+\;G(H}^{+}{)}_{\mathrm{sol}}-\;\mathrm{RTln}(V)}{\mathrm{RTln}(10)}=\frac{{\Delta G+G(H}^{+}{)}_{\mathrm{sol}}-1.8942}{1.3642}$$

in which R is the gas constant, T = 298.15 [K] is the temperature (which is used in all calculations), and RT∙ln(V) = 1.8942 [kcal mol^−1^] stands for the correction [21]

(2) $$G^{1\;[\mathrm{mol}]}{=G}^{1\;[\mathrm{atm}]}-1.8942{\;[\mathrm{kcal}\;\mathrm{mol}}^{-1}]$$

for the reference state (V = 24.46 [L], T = 298.15 [K]), from 1 [atm] to 1 [mol], whereas ∆G in [kcal mol^−1^] denotes the Gibbs free energy of the deprotonation according to the reaction

$$\mathrm{RH}\overset{\mathrm{solvent}}{⇄}R^{-}{+H}^{+}$$

The Gibbs free energy of the solvated proton G(H^+^)_sol_, is related to the solvation energy of the proton ∆G(H^+^)_sol_ by the relation [21]

(3) $${G(H}^{+}{)}_{\mathrm{sol}}=\Delta G(H^{+}{)}_{\mathrm{sol}}{+G(H}^{+}{)}_{\mathrm{gas}}$$

in which G(H^+^)_gas_ = −6.2883 [kcal mol^−1^] [22] is the free energy of a proton in the gas phase under a pressure of 1 [atm]. From (1) and (2) it follows that Equation (1) is useful evaluating p*K*_a_ using the calculated (theoretical) G(H^+^)_sol_^atm^ value, whereas G(H^+^)_sol_^mol^ = G(H^+^)_sol_^atm^ −1.8942 [kcal mol^−1^] can be compared to the experimental data. To estimate the Gibbs free energies of the solvated species involved in the deprotonation process, quantum-chemical computational methods at different levels of theory have been employed [5,6]. The calculations performed for the water medium revealed that the application of Equations (1)–(3) in the calculation of p*K*_a_ presents three primary challenges:

(i) The accuracy of p*K*_a_ reproduction is contingent upon the computational method employed to determine G(RH) and G(R^−^); it varies within an MAE (mean absolute error) range of 0.51–2.86 p*K*_a_ for monoprotic acids, depending on the method used in the calculations [5].
(ii) The value of ∆G(H^+^)_aq_, as determined by experimental [23], theoretical [24,25,26,27,28,29,30], and mixed [30] approaches, ranges from −244.9 to −266.7 [kcal mol^−1^], which significantly influences the predicted p*K*_a_ values.
(iii) Equation (1) was effectively utilized to reproduce p*K*_a_ parameters for monoprotic [5,6,7] compounds; however, its application to polyprotic molecules undergoing multi-step proton detachment yielded [4] results that deviated significantly from the experimental ones.

To address the first challenge, calculations are conducted using a range of available semi-empirical and ab initio methods, incorporating various basis sets and solvation models to identify those that most accurately reproduce experimental p*K*_a_ values [5,6]. Additionally, a linear similarity relation [5,31,32,33] such as

(4) $$pK_{a}=a\cdot pK_{a}(\mathrm{the})+b$$

is employed to enhance the accuracy of p*K*_a_ reproduction by adjusting the a and b parameters to the set of experimental p*K*_a_ = p*K*_a_(exp) data and theoretical p*K*_a_(the) values. This approach improves the accuracy of p*K*_a_ prediction from MAE = 0.51–2.86 to MAE = 0.30–0.67 in p*K*_a_ units for 22 monoprotic acids analyzed [5]. In the case of thiols, the MAE = 0.57–0.96 [32], whereas for phenols, carboxylic acids, and amines, the MAE values are lower than 0.7 p*K*_a_ units [31]. However, this level of accuracy still remains higher than the experimental error range of 0.01–0.1 in p*K*_a_ units obtained, e.g., for the multi-step deprotonation of GA [12,13]. To concurrently address the first and second challenges, Dutra et al. [6] proposed an approach wherein the average <G(H^+^)_sol_> of the G(H^+^)_sol_ values could be calculated using the equation below

(5) $${G(H}^{+}{)}_{\mathrm{sol}}=1.3642\cdot pK_{a}{\left(\exp)+G(\mathrm{RH}\right)}_{\mathrm{sol}}-{\;G(R}^{-}{)}_{\mathrm{sol}}+1.8942$$

depending on the experimental p*K*_a_(exp) and the theoretical G(RH)_sol_ and G(H^−^)_sol_ values obtained using diverse calculation methods for a specific class of compounds with similar chemical structures. This average value is subsequently incorporated into Equation (1) to form Equation (6):

(6) $$pK_{a}=\frac{{G(R}^{-}{)}_{\mathrm{sol}}-{\;G(\mathrm{RH})}_{\mathrm{sol}}+\langle {G(H}^{+}{)}_{\mathrm{sol}}\rangle -\;1.8942}{1.3642}$$

which can be used to calculate the p*K*_a_ values for all compounds within the studied class. Calculations performed using this scheme for 22 monocarboxylic acids in an aqueous medium revealed [6] that the combination of density functional theory (DFT) with the local spin density approximation (LSDA) functional provided superior performance in nearly all calculations, achieving accuracy levels comparable to those obtained using the G4CEP (Gaussian 4 Compact Effective Pseudopotential) method employed by Silva and Custodio [5]. In this study, the calculations conducted for 22 monoprotic acids using the G4CEP method, in conjunction with (i) the SMD solvation model, (ii) SMD +1 H_2_O (with one explicit water molecule), and (iii) the SMD + 1 H_2_O with a linear correction based on Equation (4) and experimental p*K*_a_(exp) values, yielded mean absolute errors of 0.83, 0.51, 0.30 in p*K*_a_ units, respectively. A comparative analysis with the results obtained by DFT at the B3LYP and BMK theory levels revealed significant errors reaching up to 2 p*K*_a_ units. In contrast, the largest deviations observed for the G4CEP method rarely exceeded 1 p*K*_a_ unit [5].

Based on the results reported above, the primary objectives of the present study were fourfold:

(i) To employ a modified version of the method proposed by Dutra et al. [6] to determine the p*K*_a_1,2,3,4 parameters for the four-stage deprotonation of GA, within the bounds of experimental errors of 0.01–0.40 p*K*_a_ units.
(ii) To calculate the Gibbs free energy of the hydrated proton G(H^+^)_aq_ and proton hydration ∆G(H^+^)_aq_ by fitting this parameter via the improved version of Equation (4) to the experimental p*K*_a_ values reported in [11,12,13,14].
(iii) To select the experimental data that most accurately represent the p*K*_a_ values for GA within the framework of the proposed approach.
(iv) To demonstrate that the accurate reproduction of the p*K*_a_ of GA, within the range of experimental errors, can be achieved using the DFT method and LSDA/QZVP level of theory combined with the SMD solvation model, without the explicit inclusion of a water molecule.

## 2. Results and Discussion

**Figure 3:**
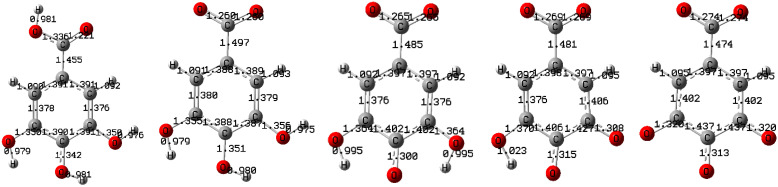
The optimized geometries of neutral GA molecule and its anionic forms GA^−N^ N = 1,2,3,4. The calculations were performed in a water medium at the LSDA/QZVP theory level, using the SMD solvation model. The values of the bond lengths are presented.

To achieve the primary objectives of this work, the geometries of the neutral GA molecule and its four anionic forms (Figure 2) were optimized in the water medium employing the DFT method at the different theory levels (LSDA, M062X, B3LYP), and the basis set by QZVP and the SMD solvation model and the reasons for their application are set out in the Materials and Methods section. The optimized geometries of the neutral GA molecule and its anionic forms generated at the LSDA/QZVP theory level are presented in Figure 3, while Table 1 and Table 2 report the corresponding Gibbs free energy values and their differences used in the determination of the p*K*_a_1,2,3,4 parameters.

**Figure 4:**
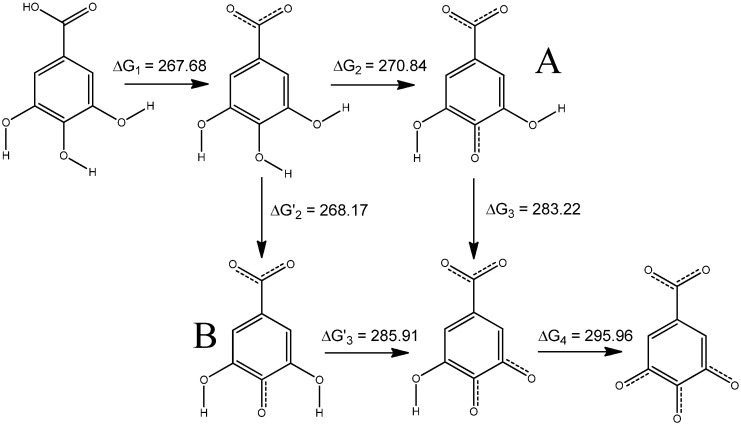
Potential scenarios for the deprotonation of GA, involving dianions A and B. The values (in [kcal mol^−1^]) of Gibbs free energies for the A reaction pathway are taken from Table S1 and Table 1, whereas ΔG’_2_ = 268.1668 and ΔG’_3_ = 285.9096 [kcal mol^−1^]. Both processes are isoenergetic as ΔG_2_ + ΔG_3_ = ΔG’_2_ + ΔG’_3_.

In this study, we examined two potential scenarios (Figure 4) for the deprotonation of GA, involving the dianion A, which possesses a higher energy, at G(GA^−2^) = −642.539452 [Ha], than its conformer B, at G(GA^−2^) = −642.544071 [Ha]. The energy disparity arises from a conformational change that contributes additional energy 2.90 [kcal mol^−1^]. The rotation barrier for an A → B transition is equal to 2.93 [kcal mol^−1^], whereas for B → A it is 5.61 [kcal mol^−1^]. These results were calculated using the free energy of the transition state [AB]^‡^ G^‡^ = −642.534128 [Ha]. Despite the energetic difference between dianions A and B, the reaction pathways for both molecules are isoenergetic as ΔG_2_ + ΔG_3_ = ΔG’_2_ + ΔG’_3_. However, incorporating the low-energy dianion B into the calculations yields ΔG’_2_ and ΔG’_3_ values that fail to accurately reproduce p*K*_a_2 and p*K*_a_3 descriptors. For this reason, all calculations were carried out using scheme A.

The calculations performed at the LSDA/QZVP theory level using the SMD solvation model indicated that the method based on Equations (5) and (6) yields an unsatisfactory reproduction of the experimental p*K*_a_ values of GA. For example, set IV provides the mean value <G(H^+^)_aq_> = −263(5) [kcal mol^−1^], exhibiting a substantial standard error, and produces p*K*_a_ = 1.77, 4.09, 13.16, 22.50. Attempts to refine these results using Equation (4) also failed to generate satisfactory agreement with the experimental p*K*_a_ (exp) values of 4.16, 8.55, 11.40, and 12.80. For the adjusted parameters a = 0.362 (125) and b = 5.47 (1.65) obtained with a standard error (SE) of 2.0479 of estimation and a coefficient of determination (R^2^) of 0.8077, the corrected p*K*_a_ values were equal to 6.11, 6.95, 10.24, 13.62. This discrepancy suggests that the relationship between the theoretical and experimental p*K*_a_ values is more complex than the linear relation (4) utilized so far. Accordingly, in this study, the linear similarity relation (4) was replaced with a non-linear self-similar formula like that shown below

(7) $$\begin{array}{l} pK_{a}N=a\cdot pK_{a}{\left(\mathrm{the}\right)}^{c}=a{\left[\frac{{\Delta G}_{N}{+G(H}^{+}{)}_{\mathrm{aq}}-1.8942}{1.3642}\right]}^{c}\\ \;\Delta G_{N}{=G(\mathrm{GA}}^{-N}{)}_{\mathrm{aq}}-{\;G(\mathrm{GA}}^{-(N-1)}{)}_{\mathrm{aq}}\;N=1,2,3,4 \end{array}$$

which simplifies to the linear relation (4) with b = 0 when c approaches 1. The parameters a, c, and G(H^+^)_aq_ in Equation (7) fitted to the four sets of available experimental GA data have subsequently been employed to reproduce the p*K*_a_1,2,3,4 parameters characterizing the four-step deprotonation of GA. The results of these calculations are reported in Table 2 and Table 3.

The results obtained reveal that the approach proposed enables both the precise determination of the Gibbs free energy of the hydrated proton G(H^+^)_aq_ = −265.7155(75) [kcal mol^−1^] and the accurate reproduction of the experimental p*K*_a_ values by taking advantage of the LSDA/QZVP theory level and set IV of the datapoints [13]. The results fall within the range of experimental errors, with MAE = 0.019 in p*K*_a_ units. As experimental data are typically represented by the Gibbs free energy of the proton hydration, this parameter can also be determined through a fitting procedure utilizing Equations (3) and (7). The findings presented in Table 4 demonstrate that parameters ∆G(H^+^)_aq_ = −259.4272(75) [kcal mol^−1^], a = 7.271(57), and c = 0.1858(31) reproduce set IV [13] of p*K*_a_ with R^2^ = 1.0000 and SE = 0.0454.

**Figure 5:**
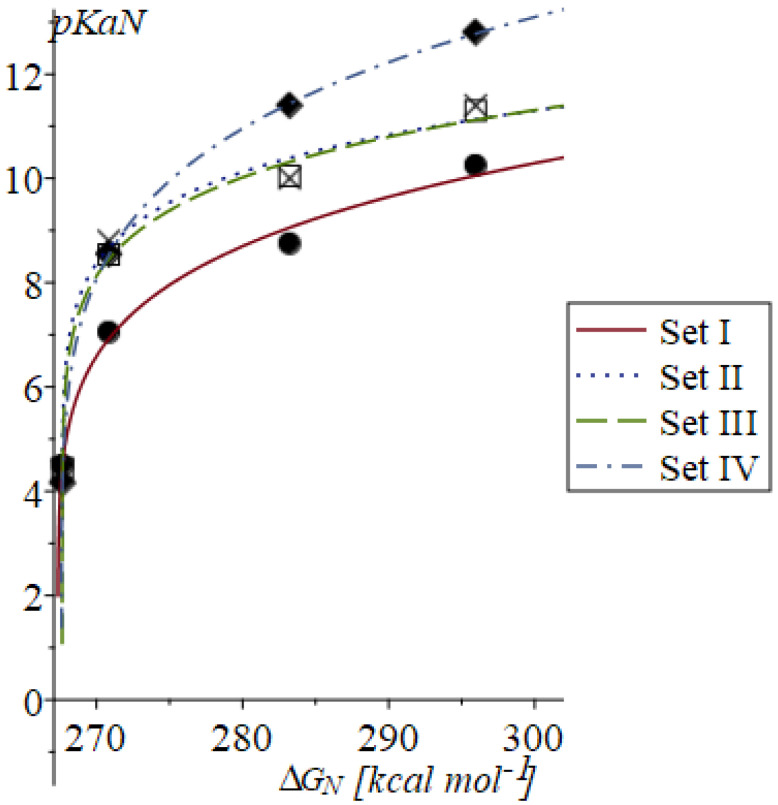
Plots of the self-similar relationship (7) with ∆G_N_ calculated at the LSDA/QZVP theory level, using the SMD model (water medium) and the four experimental datasets represented by the graphical symbols I—●; II—□; III—x; and IV—♦.

The ∆G(H^+^)_aq_ =−259.4272(75) calculated using the LSDA/QZVP theory level and set IV is consistent with the experimental value of −259.5 [kcal mol^−1^] obtained by Lim et al. [23]. This value corresponds to the average −4.5 [V] of five measurements of the standard hydrogen potential (−4.43, −4.43, −4.44, −4.48, −4.73 [V]), which allows for the determination of a range of the experimental values of ∆G(H^+^)_aq_ from −254 to −261 [kcal mol^−1^] [23]. The reproduction of the experimental p*K*_a_ values IV for GA using relationship (7) proved to be more accurate than that obtained using linear Equation (4). The parameters <∆G(H^+^)_aq_> = −258.6(7.4) [kcal mol^−1^], a = 0.36(13), and b = 5.4(1.7) reproduce set IV of p*K*_a_ with R^2^ = 0.8079 and SE = 0.7118, yielding corrected p*K*_a_ values of 5.96, 6.25, 7.38, 8.55. These results clearly indicate that, for the multivariate deprotonation of GA, the linear transformation (4) should be replaced by the self-similar formula given in Equation (7). Furthermore, the plots of the relationship linking p*K*_a_N and ΔG_N_ (Figure 5) generated for sets I, II, III, and IV highlight the strongly non-linear nature of this relation. The graphs of the p*K*_a_N as function of ∆G_N_ qualitatively have the same form for ∆G_N_ calculated at the LSDA, M062X, and B3LYP/QZVP theory levels.

In works [5,6], it was demonstrated that the accuracy of reproducing the experimental p*K*_a_ values for monoprotic acids can be enhanced through the implementation of an SMD solvation model that incorporates an additional explicitly included water molecule. Given that the model proposed in this work effectively reproduces set IV, achieving a maximum R^2^ of 1.0000, and a satisfactory MAE = 0.019, NMAE = 0.463 in p*K*_a_ units, the inclusion of the water molecule should not affect the accuracy of the calculations. To substantiate this hypothesis, the calculations were performed utilizing the scheme proposed by Kelly et al. [21], which generates a = 6.15(12), c = 0.2648(79), and ∆G(H^+^)_aq_ = −260.166(44) [kcal mol^−1^] from ∆G_1_ = 266.4521, ∆G_2_ = 268.6051, ∆G_3_ = 278.0447, and ∆G_4_ = 299.3631 [kcal mol^−1^], values that reproduce the p*K*_a_ of set IV with SE = 0.073, MAE = 0.030, and NMAE = 0.7651 in p*K*_a_ units. These results exhibit a lower degree of accuracy compared to those obtained without the inclusion of the water molecule in the calculations at the LSDA/QZVP theory level. To investigate the impact of dispersion effects on the p*K*_a_ and ∆G(H^+^)_aq_ parameters, calculations were performed employing the QZVP basis set, the SMD solvation model, and (i) the functional developed by Head-Gordon and collaborators [34], which incorporates Grimme’s dispersion model D2 [34], and the B3LYP functional combined with the D3 dispersion model. Such a combination has been recommended in [35]. The computational results, summarized in Table S2, indicate that dispersion effects have a negligible influence on the deprotonation of GA. It is noteworthy, however, that the value of the parameter ∆G(H^+^)_aq_ = −264.10(23) [kcal mol^−1^] is consistent with ∆G(H^+^)_aq_ = −264.29 [kcal mol^−1^] reported by Zhan and Dixon [27]. To evaluate the impact of basis sets on the accuracy of the reproduction of the p*K*_a_ and ∆G(H^+^)_aq_ parameters for GA, calculations were conducted at the 6311++G(d,p), aug-cc-pVQZ, QZVP/LSDA theory levels in conjunction with the SMD solvation model. The results obtained are presented in Table 5. The findings indicate that employing the QZVP basis set is not essential for accurately calculating the p*K*_a_ parameters in the scheme proposed, as the less extensive 6−311++G(d,p) basis set also yields results consistent with the experimental data. However, an analysis of the ∆G(H^+^)_aq_ parameter values reveals that the use of larger, more comprehensive basis sets is necessary to achieve result that align closely with the experimental one of ∆G(H^+^)_aq_ = −259.5 [kcal mol^−1^] reported by Lim et al. [23].

## 3. Materials and Methods

For monocarboxylic acids, the best reproduction of the p*K*_a_ values was achieved [5,6] using the composite G4CEP and DFT method in conjunction with the LSDA [36], M06-2X [37], and B3LYP [38,39] functionals, and the SMD solvation model as well as the Dunning’s [40] basis sets aug-cc-pVDZ and aug-cc-pVTZ. In light of the findings presented in [5,6], in this work, calculations were conducted utilizing the DFT method implemented in Gaussian vs. 16 software [40] in conjunction with the LSDA, M062X, and B3LYP functionals. To identify a basis set capable of accurately reproducing the Gibbs energy for GA and its anionic forms, preliminary calculations were performed on the energy of GA in an aqueous environment, employing the DFT/LSDA method and the SMD solvation model, utilizing basis sets cc-pVQZ, aug-cc-pVQZ [40], and QZVP [41]. The resulting energy values (in [Ha]: E_1_(cc-pVQZ) = −643.496286; E_2_(aug-cc-pVQZ) = −643.501176; E_3_(QZVP) = −643.511057), and their corresponding differences (in [kcal mol^−1^]: ∆E_12_ = 3.0685; ∆E_23_ = 6.2004; ∆E_13_ = 9.2689), guided the selection of the quadruple zeta valence polarized (QZVP) basis set [41] for subsequent calculations, as it yielded the lowest energy value for the GA. A similar situation was observed for the M062X and B3LYP functionals; consequently, the DFT method at the LSDA, M062X, and B3LYP/QZVP level of theory, combined with the SMD solvation model, was employed for the primary calculations. The input structures were constructed by taking advantage of the Gauss View-6.1 graphical interface [42], and calculations were carried out using the Gaussian version 16 A software [42,43]. Since the computations for the compounds studied at the LSDA,M062X,B3LYP/QZVP level are time consuming and their convergence depends on the initial geometry input, the optimization was divided into two stages: (i) the determination of an approximate geometry at the LSDA,M062X,B3LYP/cc-pVQZ theory levels in the gas phase, and (ii) the final calculation at the LSDA,M062X,B3LYP/QZVP theory levels in the water medium using the SMD solvation model. Thus, optimization was performed at each stage, with the geometry determined at the lower level serving as the starting point for optimization at the higher level. The optimized structures of the compounds considered in this work are displayed in Figure 3. The parameters a, c, G(H^+^)_aq_, and ∆G(H^+^)_aq_ were fitted to the four sets of experimental p*K*_a_ values for GA, including set I [4.50, 7.05, 8.75, 10.25] [11], set II [4.4(1), 8.8(1), 10.0(1), 11.4(1)] [12], set III [4.44, 8.54, 10.05, 11.3] [14], and set IV [4.16(2), 8.55(1), 11.4(1), 12.8(4)] [13]. The calculations were performed by taking advantage of Sigma Plot vs. 11 software—the results of calculations together with indicators of goodness of the fit, R^2^, coefficient of determination, and SE, standard error of estimation, are presented in Table 2 and Table 4. The fitted parameters were utilized to calculate the p*K*_a_N values for GA, as presented in Table 3 and Table 4, along with the mean absolute errors (MAEs) and the normalized mean absolute errors (NMAEs) associated with the reproduction of the experimental p*K*_a_N(exp) data, defined as follows

(8) $$\mathrm{MAE}=\frac{1}{4}\sum_{N=1}^{4}\left|pK_{a}N\text{-}pK_{a}N(\exp)\right|\;\;\mathrm{NMAE}=\frac{1}{4}\sum_{N=1}^{4}\frac{\left|pK_{a}N\text{-}pK_{a}N(\exp)\right|}{u_{N}}$$

Here, u_N_ denotes experimental errors in the measurements provided for datasets II (u_N_ = 0.1) [12] and IV (u_1_ = 0.02, u_2_ = 0.01, u_3_ = 0.10, u_4_ = 0.40) [13]. A value of NAME ≤ 1 indicates the reproduction of experimental data within the accuracy of the measurement error.

## 4. Conclusions

The proposed method for determining the p*K*_a_ parameters of GA enables the simultaneous validation of both the method used and the experimental data employed in the calculations. Among the four datasets analyzed, the non-linear model demonstrated the best agreement with dataset IV [13], which exhibited the highest statistical consistency when reproduced using the DFT method at the LSDA/QZVP theory level and the SMD solvation model. The parameters presented in Table 3 reproduce experimental dataset IV with a standard error, SE, of 0.0454 and a coefficient of determination, R^2^, of 1.0000, achieving reproduction of the experimental p*K*_a_ values [13] for GA within the limits of experimental errors as NMAE = 0.463 p*K*_a_ units. These findings confirm the validity of the self-similar Formula (7) in particular and the overall calculation methodology in general. Additionally, the reproduction of the experimental p*K*_a_ values for GA using relationship (7) has been proved to be more accurate than that obtained with Equation (4). The results obtained indicate that, for the multivariate deprotonation of GA, the linear transformation (4) should be replaced by the self-similar formula given in Equation (7). The application of Equation (7) in conjunction with the ΔG_N_ values calculated using the DFT method at the LSDA/QZVP theory level, alongside the SMD solvation model, is sufficient to achieve a satisfactory reproduction of the measured p*K*_a_ values of GA. Thus, the utilization of the computationally time-consuming G4CEP method and the SMD model with the inclusion of an additional water molecule, as suggested in works [5,6], is deemed unnecessary for this objective. It is acknowledged that the proposed approach represents a significant simplification of the real deprotonation process due to the complex reactions that occur between GA and water. These reactions result in substantial modifications to the structure of GA [44] and its anionic forms, which in turn have a pronounced effect on the kinetics and thermodynamics of GA deprotonation. The proposed model is not derived from the first principles but constitutes a quasi-phenomenological framework, wherein the semi-empirical parameters a and c encapsulate all *hidden variables* that are not explicitly incorporated into the analysis. A first-principles model should, at the molecular level, account for all possible structures of GA, including polymorphs, rotamers, and tautomers and their anionic forms, as well as their interactions with solvent molecules and the formation of complex polymolecular systems. It will be essential to undertake research in this regard in the near future.

## Figures and Tables

**Table 1:** The differences ∆G_N_ [kcal mol^−1^] in Gibbs free energies of neutral GA^0^ molecule and its anionic forms GA^−N^ N = 1,2,3,4 including the zero-point energy corrections from Table S1. The calculations were performed in the water medium at the LSDA, M062X, and B3LYP/QZVP theory levels, using the SMD solvation model.

	∆G_N_ [kcal mol^−1^]	LSDA	M062X	B3LYP
∆G_1_	G(GA^−1^)_aq_–G(GA^0^)_aq_	267.6773	270.6210	272.7539
∆G_2_	G(GA^−2^)_aq_–G(GA^−1^)_aq_	270.8450	279.5730	280.5099
∆G_3_	G(GA^−3^)_aq_–G(GA^−2^)_aq_	283.2258	289.4136	290.8582
∆G_4_	G(GA^−4^)_aq_–G(GA^−3^)_aq_	295.9617	297.0059	298.7058

**Table 2:** The values of the parameters a, c (dimensionless), and G(H^+^)_aq_ ([kcal mol^−1^]) fitted via Equation (7) to the four sets of experimental p*K*_a_ for GA reported in [11,12,13,14]. The indicators of goodness of the fit/standard error (SE) of estimation and the coefficient of determination, R^2^, are presented.

	LSDA	M062X	B3LYP	LSDA	M062X	B3LYP
Parameter	Set I			Set II		
G(H^+^)_aq_	−265.47(32)	−62.1(3.2)	−266.6(2.5)	−265.775(20)	−268.12(65)	−270.51(44)
a	5.87(59)	2.02(80)	2.84(94)	7.87(64)	5.36(48)	5.90(96)
c	0.177(38)	0.51(11)	0.41(10)	0.114(33)	0.247(66)	0.216(61)
SE	0.3861	0.2031	0.2630	0.4959	0.4020	0.4383
R^2^	0.9918	0.9977	0.9962	0.9910	0.9941	0.9930
**Parameter**	**Set III**			**Set IV**		
G(H^+^)_aq_	−265.764(26)	−267.78(51)	−270.29(38)	−265.7155(75)	−266.81(45)	−269.63(15)
a	7.59(83)	4.91(57)	5.48(60)	7.271(57)	3.60(34)	4.33(16)
c	0.126(23)	0.275(41)	0.240(40)	0.1858(31)	0.420(31)	0.364(13)
SE	0.3327	0.2261	0.2666	0.0454	0.1453	0.0766
R^2^	0.9959	0.9981	0.9973	1.0000	0.9995	0.9994

**Table 3:** The values of p*K*_a_1,2,3,4 reproduced by Equation (7) with parameters from Table 1 and Table 2 evaluated for the four sets of experimental p*K*_a_ data reported in [11,12,13,14]. The mean absolute error (MAE) of the data reproduction is presented.

	p*K*_a_ (exp)	p*K*_a_			p*K*_a_ (exp)	p*K*_a_		
Parameter	Set I	LSDA	M062X	B3LYP	Set II	Set I	LSDA	M062X
p*K*_a_1	4.50	4.52	4.52	4.52	4.4(1)	4.61	4.40	4.40
p*K*_a_2	7.05	6.92	6.95	6.93	8.8(1)	8.67	8.67	8.67
p*K*_a_3	8.75	9.06	8.91	8.96	10.0(1)	10.40	10.32	10.35
p*K*_a_4	10.25	10.05	10.17	10.14	11.4(1)	11.13	11.20	11.17
MAE		0.165	0.090	0.115		0.253	0.163	0.178
**Parameter**	**Set III**	**LSDA**	**M062X**	**B3LYP**	**Set IV**	**LSDA**	**M062X**	**B3LYP**
p*K*_a_1	4.44	4.55	4.44	4.44	4.16(2)	4.161	4.155	4.168
p*K*_a_2	8.54	8.45	8.46	8.46	8.55(1)	8.536	8.607	8.600
p*K*_a_3	10.05	10.32	10.23	10.26	11.40(10)	11.44	11.28	11.37
p*K*_a_4	11.30	11.12	11.19	11.16	12.80(40)	12.78	12.87	12.87
MAE		0.163	0.118	0.108		0.019	0.063	0.060

**Table 4:** The values of the parameters a, c (dimensionless), and ∆G(H^+^)_aq_ ([kcal mol^−1^]) fitted to the four sets of experimental p*K*_a_ for GA via Equation (7) including Equation (3) and ∆G_N_ calculated at the LSDA/QZVP theory level using the SMD solvation model and a water medium. The indicators of goodness of the fit/standard error (SE) of estimation and the coefficient of determination (R^2^) are presented. The mean absolute error (MAE) and the normalized mean absolute error (NMAE defined in Equation (8)) of the reproduced p*K*_a_N parameters are presented.

Parameter	Set I	Set II	Set III	Set IV
∆G(H^+^)_aq_	−259.19(32)	−259.486(20)	−259.476(26)	−259.4272(75)
a	5.87(59)	7.871(39)	7.59(43)	7.271(57)
c	0.177(38)	0.114(33)	0.126(23)	0.1858(31)
R^2^	0.9918	0.9910	0.9959	1.0000
SE	0.3861	0.4959	0.3327	0.0454
p*K*_a_1	4.51	4.40	4.44	4.16
p*K*_a_2	6.92	8.67	8.45	8.54
p*K*_a_3	9.08	10.40	10.32	11.44
p*K*_a_4	10.05	11.13	11.12	12.78
MAE	0.160	0.199	0.134	0.019
NMAE		1.992		0.463

**Table 5:** The effect of basis set on the theoretical reproduction of proton hydration energy ∆G(H^+^)_aq_ and p*K*_a_N parameter values. The calculations were performed in the water medium at the LSDA/QZVP theory levels, using the SMD solvation model. The experimental value of ∆G(H^+^)_aq_ = −259.5 [kcal mol^−1^] was reported by Lim et al. [23].

	6311++G(d,p)	aug-cc-pVQZ	QZVP
∆G(H^+^)_aq_	−257.1671(51)	−259.2358(72)	−259.4272(75)
a	7.156(34)	7.268(54)	7.271(57)
c	0.1934(19)	0.1869(29)	0.1858(31)
R^2^	1.0000	1.0000	1.0000
SE	0.0265	0.0430	0.0454
p*K*_a_1	4.159	4.160	4.161
p*K*_a_2	8.542	8.537	8.536
p*K*_a_3	11.423	11.435	11.436
p*K*_a_4	12.788	12.778	12.776
MAE	0.011	0.017	0.019
NMAE	0.268	0.427	0.463

## Data Availability

All new data created are reported in this work.

## Supplementary Materials

The following supporting information can be downloaded at: https://www.mdpi.com/article/10.3390/molecules30030742/s1, Table S1. The Gibbs and zero-point energies [Ha] of neutral GA^0^ molecule and its anionic forms GA^−N^ N = 1,2,3,4 calculated in the water medium at the LSDA, M062X, B3LYP/QZVP theory levels, using the SMD solvation model. Table S2. The effect of dispersion on the theoretical reproduction of proton hydration energy ∆G(H^+^)_aq_ and p*K*_a_N parameter values. The calculations were performed in a water medium at the wB97XD/D2//QZVP, B3LYP/D3/QZVP theory levels, using the SMD solvation model. The theoretical value of ∆G(H^+^)_aq_ = −264.29 [kcal mol^−1^] is reported by Zhan and Dixon [27]. For comparison, the results obtained at the B3LYP/QZVP level of theory without taking the dispersion effect into account are shown.
