# Supplementary material for: Computation of the pKa Values of Gallic Acid and Its Anionic Forms in Aqueous Solution: A Self-Similar Transformation Approach for Accurate Proton Hydration Free Energy Estimation
Source: Molecules. 2025 Feb 6;30(3):742. doi: 10.3390/molecules30030742 (PMC11820028; doi:10.3390/molecules30030742)
Supplement: Supplementary file 1 [file molecules-30-00742-s001.zip › molecules-3438081-supplementary.pdf]

Supplementary Materials

Computation of p*K*a values of gallic acid and its anionic forms in aqueous solution: a self-similar transformation approach for accurate proton hydration free energy estimation

Marcin Molski

Quantum Chemistry Department
Faculty of Chemistry
Adam Mickiewicz University
ul. Uniwersytetu Poznańskiego 8, 61-614 Poznań, Poland
mamolski@amu.edu.pl

DFT	LSDA	M062X	B3LYP	LSDA	M062X	B3LYP
	Gibbs Free Energy [Ha]			Zero-Point Energy [Ha]		
$G(GA^0)_{aq}$	-643.422706	-646.506126	-646.741787	0.124482	0.128589	0.126738
$G(GA^{-1})_{aq}$	-642.983693	-646.061803	-646.294158	0.112040	0.115528	0.113770
$G(GA^{-2})_{aq}$	-642.539452	-645.603903	-645.834900	0.099418	0.103156	0.101533
$G(GA^{-3})_{aq}$	-642.075476	-645.129860	-645.358642	0.086791	0.090323	0.088787
$G(GA^{-4})_{aq}$	-641.591708	-644.643124	-644.869168	0.074668	0.076896	0.075331

Table S1. The Gibbs and zero-point energies [Ha] of neutral GA^0 molecule and its anionic forms GA^{-N} N=1,2,3,4 calculated in the water medium at the LSDA, M062X, B3LYP /QZVP theory levels, using the SMD solvation model.

	WB97XD/D2		B3LYP/D3		B3LYP	
**N**	**ΔG_N**	**pK_aN**	**ΔG_N**	**pK_aN**	**ΔG_N**	**pK_aN**
1	274.0848	4.157	273.1718	4.157	272.7539	4.158
2	282.7934	8.580	281.1832	8.589	280.5099	8.578
3	293.0068	11.338	291.2949	11.316	290.8582	11.338
4	300.3298	12.838	299.1043	12.85	298.7058	12.836
MAE		0.033		0.044		0.032
NMAE		0.962		1.268		0.905
$\Delta G(H^+)_{aq}$	-264.10(23)		-263.62(24)		-263.34(16)	
a	3.71(18)		4.15(23)		4.32(16)	

c	0.411(16)	0.377(19)	0.364(13)
R^2	0.9999	0.9997	0.9999
SE	0.0785	0.1047	0.0766

Table S2. The effect of dispersion on the theoretical reproduction of proton hydration energy $\Delta G(H^+)_{aq}$ and pK_aN parameter values. The calculations were performed in the water medium at the wB97XD/D2//QZVP, B3LYP/D3/QZVP theory levels, using the SMD solvation model. The theoretical value of $\Delta G(H^+)_{aq} = -264.29$ [kcal mol^{-1}] is reported by Zhan and Dixon [27]. For comparison, the results obtained at the B3LYP/QZVP level of theory without taking the dispersion effect into account are shown.
